# Choosing the Right Partner for Medication Related Osteonecrosis of the Jaw: What Central European Dentists Know

**DOI:** 10.3390/ijerph18094466

**Published:** 2021-04-22

**Authors:** Emanuel Bruckmoser, Miriam Palaoro, Lukas Latzko, Dagmar Schnabl, Sabrina B. Neururer, Johannes Laimer

**Affiliations:** 1 Private Practice for Oral and Maxillofacial Surgery, A-5020 Salzburg, Austria; research@bruckmoser.info; 2 University Hospital for Craniomaxillofacial and Oral Surgery, Medical University Innsbruck, A-6020 Innsbruck, Austria; miriampalaoro@gmail.com (M.P.); lukas.latzko@tirol-kliniken.at (L.L.); 3 Department of Operative and Prosthetic Dentistry, Medical University Innsbruck, A-6020 Innsbruck, Austria; dagmar.schnabl@tirol-kliniken.at; 4 Department of Medical Statistics, Informatics and Health Economics, Medical University of Innsbruck, A-6020 Innsbruck, Austria; S.Neururer@tirol-kliniken.at; 5 Department of Clinical Epidemiology, Tyrolean Federal Institute for Integrated Care, Tirol Kliniken GmbH, A-6020 Innsbruck, Austria

**Keywords:** medication-related osteonecrosis of the jaw, MRONJ, antiresorptive treatment, bisphosphonates, denosumab, bone metastases, dental oncology

## Abstract

Medication-related osteonecrosis of the jaw (MRONJ) is a side effect of antiresorptive drugs. In this online survey, the awareness and knowledge of dentists regarding MRONJ was evaluated, and potential implications for oncologists are discussed. Questionnaires were emailed to dentists from Germany, Austria, Switzerland, and South Tyrol to evaluate disease-related knowledge and management. In addition to the overall score, a separate score was calculated for knowledge (maximum score: 15 points) and management (maximum score: 6 points) questions, and 1197 valid replies with completed questionnaires were received. The mean overall score was 10.45 ± 3.97 points, the mean knowledge score was 7.68 ± 3.05 points, and the mean management score was 2.76 ± 1.77 points. Factors influencing the outcome of the overall score were age, specialization, continuous professional education, and the number of dental screening exams in patients before antiresorptive therapy. Due to the considerable lack of knowledge regarding MRONJ among dentists, MRONJ patients and subjects at risk should be guided towards specialists for dental screening, treatment, and follow-up. This is important from an oncologic point of view to avoid any delay for treatment start of antiresorptives, and to reveal a potentially emerging osteonecrosis at an early stage, thus, avoiding the need for interruption or even cancellation of antiresorptive therapy.

## 1. Introduction

Medication-related osteonecrosis of the jaw (MRONJ) is a potentially severe side effect of mainly antiresorptive drugs used in tumor patients with osseous metastases, multiple myeloma, as well as primary and secondary osteoporosis. Key features of this condition include areas of exposed necrotic jaw bone, pain, infection, and various complications depending on the stage of the disease [1]. This condition has become a growing problem over the past almost two decades not only for oral surgeons and dentists, but also for oncologist and other colleagues taking care of oncologic patients, such as gynecologists, urologists, and general surgeons.

The risk of developing MRONJ lies between 0.01% and 0.03% in osteoporotic, and between 1.3% and 1.8% in oncologic patients [2]. These overall incidence figures may underestimate the risk to develop MRONJ, which is greatly influenced by variables, such as drug type (low versus high potent bisphosphonates or denosumab), administration route (greater risk for i.v. compared to oral application), cumulative dose (increasing risk with longer duration), and dental surgery (see below).

With the broad use and application of highly potent bisphosphonates like zoledronic acid from the early 2000s on, and the emergence of the monoclonal antibody denosumab in 2009, the incidence of MRONJ has risen rapidly in recent years. Due to additional drugs having been identified to potentially cause MRONJ too, the incidence of this condition is expected to rise even further in the near future. Moreover, the growing number of drugs potentially causing MRONJ [3,4,5,6] emphasizes the importance of a well-designed medical history form to be completed by each patient on first visit in order to identify patients at risk.

To reduce the risk for the development of MRONJ, a dentoalveolar focus screening before the initiation of any antiresorptive therapy should always be performed [1]. It is a common misconception that only dentate patients should undergo such a screening procedure. Moreover, in edentulous subjects, alveolar pathologies may be present, which often can only be seen in a panoramic radiograph (e.g., jaw cysts). On the occasion of the focus screening appointment, dentures should be inspected and, if necessary, relined to avoid the development of pressure sores potentially evolving to MRONJ lesions [7,8,9].

Dental surgical procedures such as tooth extractions during antiresorptive treatment are known to significantly increase the risk for MRONJ, especially in oncologic patients [10]. For this reason, dentoalveolar interventions including tooth removal, cystectomy, root tip resection, etc., should be avoided if possible. If unavoidable, oral surgery needs to be performed under strict conditions including pre- and post-operative antibiotics, removal of bony spurs followed by closure of the extraction wound with a tension-free flap after periosteal relieving incisions. Even simple appearing procedures like an uncomplicated tooth extraction must not be performed as usual due to the before mentioned special requirements.

In recent years, several authors evaluated the influence of antiresorptive drugs on various cells and different cellular types including human gingival fibroblasts [11] and human periodontal ligament stem cells [12]. Moreover, the effect of bisphosphonates on the osteogenic activity of osteoprogenitor cells cultured on titanium surfaces has been investigated [13]. Further, innovative therapeutic approaches have been evaluated including fluorescence-guided bone surgery [14], ozone [15], advanced-platelet rich fibrin (A-PRF) and injectable-platelet rich fibrin (i-PRF) [16], and laser combined with platelet-rich plasma [17]. Discoveries on oral mesenchymal stem cell-derived exosomes have been reported in a recent review [18].

### Study Aim

The purpose of this online survey was to evaluate awareness and knowledge in dentists regarding MRONJ, and to discuss potential implications for oncologists.

## 2. Materials and Methods

For this online cross-sectional study, an electronic questionnaire including 14 questions was designed using the software REDCap (Research Electronic Data Capture, Vanderbilt University, Nashville, TN, USA) [19]. REDCap is a web-based application to support clinical and translational research. It is easy to implement and enables an individual design of data acquisition tools using a point-and-click approach. Apart from the usual data collection fields (text, drop-down, etc.), the software provides calculated fields and skip logic too, thereby enabling the implementation of dependent fields and supporting the dynamic design process of the electronic questionnaire [20]. Completed data fields are shown to the participant. For the statistical analysis, a separate code is saved in the database (e.g., 0 = no, 1 = yes). Using the data export tool, electronic data including context information can be imported into various statistical software, such as SPSS 26 (IBM Corp., Armonk, NY, USA).

To send out the electronic questionnaire, publicly available e-mail addresses were retrieved from various sources including the yellow pages and the regional dental associations. Following this, the respective dentists in Austria, Germany, Switzerland, and South Tyrol were contacted via e-mail in January 2019.

Regarding the recruitment process, all dentists in the before-mentioned countries were potentially eligible for inclusion into this online survey irrespective of the place of work (hospital, clinic, dental office, or a combination thereof).

Since the questionnaire was not intended for use in patients but for colleagues only (i.e., dentists), ethical approval was not required. This was confirmed in written form by the responsible academic authorities of the Medical University Innsbruck, Austria (legal department, data protection supervisor, and vice rector for finance and IT).

In order to provide a clearly structured analysis, all questions and outcomes were regrouped into three categories: six general questions concerning demographics and daily practice (G1–6), four questions assessing specific knowledge about MRONJ (“knowledge questions”, K1–4), and four questions evaluating the dentists’ competence regarding the management of MRONJ patients (“management questions”, M1–4). All questions (translated into English) are listed in Table 1.

Questions G1, G2, G4–6, K4, and M1–3 were single-choice, whereas for questions G3, K1-K3, and M4 multiple answers were correct or allowed. For question M4, answers 2–4 were correct. However, dentists ticking the box for “I don’t treat such patients by myself” were analyzed separately since this choice does not represent a wrong answer. Questions G1–6 were purely informative (age, working place, services offered, etc.) with no right or wrong answers. Evaluative questions (right versus wrong) comprised four single-choice (K4, M1–3) and four multiple-choice questions (K1–3, M4). Each question (or part of a question) answered correctly was rated as correct yielding one score point. There were no deductions for wrong answers. Scores were calculated by summing up the correct answers for “knowledge”, “management”, and “overall”. The maximum reachable score for the “knowledge questions” (K1–4) was 15; the maximum for the “management questions” (M1–4) was 6. Thus, the maximum overall combined score was 21 points. Questions G1–6 were assumed to have potential influence on the knowledge and/or management and/or overall score, which was evaluated by appropriate statistical tests (see below).

For all variables of interest, sources of data (including measurements) were the electronically returned questionnaires completed by the participants.

There was no calculation of study size since the aim of this survey was to get feedback from as many dentists as possible in the Central European germanophone region.

### Statistical Methods

Data is either presented as mean ± standard deviation or absolute and relative frequencies. Chi-square tests were performed to analyze categorical data, while Student’s *t*-tests were used for assessing group differences of interval and ratio scaled data. 

In order to facilitate the comparison of the retrieved items of the questionnaire, two subscores were calculated and analyzed. These subscores were summarized into an overall score. In order to analyze these scores, logistic regression models were calculated. Due to the high number of respondents, available-case analysis was given preference over complete-case analysis.

All statistical analyses were performed using SPSS 26 (IBM Corp. Released 2019. IBM SPSS Statistics for Windows, Version 26.0. Armonk, NY, USA). *p*-values < 0.05 were considered statistically significant.

## 3. Results

The questionnaire was sent out to a total of 25,410 dentists. In 1216 cases, mail delivery was not successful (error message) leaving 24,194 successfully delivered emails containing the link required for completion of the questionnaire. 1473 replies were registered, however, 276 dentists did not tick the box regarding the data privacy statement. Hence, 1197 completed questionnaires could be further analyzed corresponding to a response rate of 4.9%. In Table 1, “*n* =” refers to the total of valid responses and is indicated for each question. 

Responses to demographic and general questions (G1–6) are provided in the first part of Table 1. Regarding the third question (services offered), it is interesting to note that 105 colleagues (8.8%) were apparently specialized in oral surgery since they did only indicate this field of expertise. This information cannot be drawn from Table 1 alone because multiple answers were allowed.

Participating dentists (*n* = 1170) scored 10.45 points on average (mean) with a standard deviation (SD) of ±3.97 for the overall score. The mean of the knowledge score summing up responses to questions K1–4 was 7.68 ± 3.05 points (*n* = 1180). The mean of the management score summing up M1–4 was 2.76 ± 1.77 points (*n* = 1178).

The results of the inferential statistical analyses are depicted in Table 2, where significant *p*-values are highlighted in bold. The following variables had a statistically significant influence on the outcome of the overall score comprising both knowledge and management questions: age (the younger the better), oral surgery (the more oral surgery the better), continuous professional education (the more the better), and the number of dental screening exams before antiresorptive therapy (the more patients the better). The following variables had a statistically significant influence on the outcome of the knowledge score: age (the younger the better), oral surgery (the more oral surgery the better), continuous professional education (the more the better), and the number of dental screening exams before antiresorptive therapy (the more patients the better). The following variables had a statistically significant influence on the outcome of the management score: oral surgery and conservative dentistry/prosthetics (the more the better), continuous professional education (the more the better), and the number of dental screening exams before antiresorptive therapy (the more patients the better).

## 4. Discussion

We report the first online study to evaluate awareness and knowledge regarding MRONJ in the Central European community of germanophone dentists including Germany, Austria, Switzerland, and South Tyrol (Italy). The overall outcome in this online survey (10.45 out of 21 points in the overall score) showed considerable deficiencies. From an oncological point of view, patients with bone metastases requiring antiresorptive treatment should be referred to specialists or even specialized MRONJ clinics for dental screening prior to any therapy with bisphosphonates or denosumab. Even patients at risk for bone metastases (e.g., in breast cancer, prostate cancer, etc.) should be referred in a timely manner to avoid any treatment delay in case of emerging osseous metastases. Collaborations between oncologists and oral surgeons need to be established and strengthened so that every oncologist has a dental expert or clinic to address any requests or referrals to [8].

Since the before mentioned approach is very resource intensive and may introduce barriers to care, the important role of improving dental education (both undergraduate and continued professional development) in better training the dental workforce in management of MRONJ should be emphasized. Building up a well-trained dental community would offer a broadly available and low-threshold first point of contact. Well-trained dentists would also be able to better assess which cases they can handle alone and which patients need to be referred to specialists or even specialized MRONJ clinics. Finally, dental office based colleagues would be an essential basic contact for the considerable number of osteoporosis patients suffering from MRONJ.

Regarding influencing factors with statistical and clinical significance, it does not come as a surprise that colleagues mainly (or exclusively) performing oral surgery demonstrated a high competency in this online survey. The management of MRONJ often includes surgical procedures so that colleagues performing a lot of oral surgery are presumably more confident and experienced when it comes to MRONJ treatment. Once more, this finding points out the importance of competent and experienced partners where oncologists can refer their patients to, be it for dental screening or for surgical treatment in cases where MRONJ has already developed due to the application of antiresorptives for management of bone metastases. Failure to do so may result in progression of MRONJ which is much more difficult to handle compared to early stage treatment [21,22].

The number of dental screening exams prior to antiresorptive treatment performed per year significantly influenced the outcome of all scores (knowledge, management, and overall score) in a positive way. This may not be a big surprise either but, once again, underlines the fact that MRONJ patients should be guided towards specialized oral surgeons who manage such cases on a regular basis. As already discussed redundantly, systematic screening for dentoalveolar pathologies is of utmost importance prior to any antiresorptive therapy to lower the risk for MRONJ.

The influence of the participants’ age on the outcome could be due to the fact that MRONJ has been known for less than 20 years. This highlights the importance of continuous professional education, especially for older colleagues who have not heard of this drug side effect during their studies in dental school. This is further supported by the finding that continuous professional education had a significantly positive influence on the outcome in our online survey.

There are several limitations to this online survey. Although we have considered all available sources to retrieve the maximum of email addresses, our final database containing 24,194 valid email addresses is certainly not complete. Furthermore, there are—presumably rather few—colleagues who may not have an email account at all, which automatically excluded them from being contacted. No efforts were made to contact dentists by conventional mail, which represents an inclusion bias in any online survey. Since the older generation showed a worse outcome, we believe that inclusion of these dentists might have yielded an even more pronounced result with regard to the influence of age on the score results.

Another shortcoming is undoubtedly the low response rate of 4.9%. The main issue in this context is the question whether our study sample can still be regarded as representative. We do not see a reason why one or the other group of dentists would have been more likely to respond or to ignore our invitation to participate. However, this assumption remains speculative, as it is not supported by further evidence.

An inherent limitation of any online survey of this kind is the fact that no time limit was imposed, and that there was obviously no control of the participating dentists during completion of the questionnaire. This means that participants could have potentially consulted a variety of resources such as books, journals, online content, etc. However, since this survey was fully anonymous without any consequences for the participants’ personal life or professional activities, we are convinced that this issue should not have skewed the results significantly.

Although this is the first online survey to evaluate the awareness and competence regarding knowledge and management of MRONJ in the Central European community of germanophone dentists, there have been a few studies in other countries published over the past years.

The first study of this kind was conducted in Ontario, Canada [23], in which 1579 responses to a web-based questionnaire in a random sample of dentists were statistically analyzed. Sixty percent had a good knowledge of bisphosphonates and related osteonecrosis of the jaw. However, only 23% followed the respective guidelines for surgical treatment. Sixty-three percent indicated that they would rather refer patients taking bisphosphonates, and about 50% did not feel comfortable treating osteonecrosis patients.

In a recent study conducted in Brazil [24], 1032 dentists, 239 physicians, and 99 nurses were asked to complete a questionnaire at a Brazilian hospital as well as on the occasion of the International Congress of Dentistry in Brazil and the Brazilian Congress of Oral Medicine and Oral Pathology. In the group of dentists and physicians, training time had a significant impact on MRONJ knowledge. Dentists who were specialized in stomatology, oral and maxillofacial surgery, and special care dentistry showed a significantly better outcome.

Another study [25], which was conducted in India, included graduates, postgraduates, and faculty members from six dental schools. The self-administered questionnaire was prepared using a Google form accessible through a link, which was sent out via email, and 234 responses were received and statistically analyzed. Most participants were aware of the term “MRONJ” (83.3%), indications for bisphosphonates (61.5%), and their mechanisms of action (72.2%). Lack of knowledge regarding the concept of “drug holiday” and regarding risk factors for MRONJ was relatively high (68.4% and 61.5%, respectively).

A study from Saudi Arabia [26] used questionnaire forms distributed in soft copies using Google forms. The final sample comprised 74 dentists of which 60.8% knew about MRONJ, and 79.7% had never seen an MRONJ patient. Only 18.9% were aware of the relationship between the risk of MRONJ in osteoporotic patients and long-term (>4 years) use of bisphosphonates, and 59.5% of the participants believed that radiotherapy could cause MRONJ.

In another study from Saudi Arabia [27], statistical analysis of 607 responses to self-administered questionnaires comprising close-ended questions showed insufficient knowledge regarding MRONJ. Only 70% of the participants had heard about MRONJ, and less than 50% were aware of risk factors and clinical features of this condition. Specialists performed better than general dentists.

Overall, it is difficult to compare studies among each other and with our own work. There are no international standards with regard to structure and content of such questionnaires. As outlined in the previous paragraphs, there are significant regional differences to consider. However, a general tendency could be noted that awareness, knowledge, and competence regarding the management of MRONJ is not considered satisfactory by most authors, which is in line with our own findings.

## 5. Conclusions

In this comprehensive online survey of dentists in Central Europe, considerable deficiencies were revealed. Younger dentists and colleagues with a focus on oral surgery performed better in this questionnaire-based study. Continuous professional education and a high number of dental focus screening exams performed (prior to antiresorptive therapy) significantly favored a better outcome. From an oncological point of view, it is important to know the right partners (specialized dentists, oral surgeons, MRONJ clinics) where the respective patients can be referred. This does not only include dental screening exams prior to initiation of antiresorptive treatment but also MRONJ therapy and follow-up visits on a regular basis. These recall exams are key to maintaining good oral health in this patient collective and to immediately take action in case of an emerging jaw osteonecrosis.

## Figures and Tables

**Table 1:** General (G1–6), knowledge (K1–4), and management (M1–4) questions of the online survey.

	Questions	Answers	Absolute Frequency	Relative Frequency (%)
G1	How old are you? (*n* = 1189)	<35 years	152	12.8
		36–45 years	254	21.4
		46–55 years	350	29.4
		56–65 years	364	30.6
		>65 years	69	5.8
G2	Where do you work? (*n* = 1189)	Dental office/private practice	1105	93
		Hospital or dental clinic	30	2.5
		both	54	4.5
G3	What range of services do you offer on a routine basis? (*n* = 1197)	Conservative dentistry and prosthetics	1056	88.2
		Oral surgery	891	74.4
		Orthodontics	195	16.3
G4	How much oral surgery do you perform per day? (*n* = 1188)	0%	42	3.5
		<5%	336	28.3
		6–25%	578	48.7
		26–50%	120	10.1
		>50%	112	9.4
G5	Have you ever attended a seminar, course, meeting, conference, etc. about osteonecrosis of the jaw? (*n* = 1188)	yes	876	73.7
G6	How many patients for dental focus screening before antiresorptive therapy do you see per year? (*n* = 1189)	0	231	19.4
		1–5	587	49.4
		6–10	223	18.8
		11–15	52	4.4
		>15	96	8
K1	Which of the following terms do you know? (*n* = 1197)	BRONJ	570	47.6
		ARONJ	243	20.3
		MRONJ	318	26.6
		ONJ	552	46.1
K2	Which of the following drugs do you think can potentially cause osteonecrosis of the jaw as a side effect? (*n* = 1197)	Prolia ^®^	716	59.8
		Sutent ^®^	197	16.5
		Zometa ^®^	964	80.5
		XGEVA ^®^	513	42.9
		Avastin ^®^	416	34.8
K3	For which of the following conditions do you think patients are likely to get drugs potentially causing osteonecrosis of the jaw? (*n* = 1197)	Breast cancer	899	75.1
		Prostate cancer	649	54.2
		Multiple myeloma	380	31.7
		Osteoporosis	1051	87.8
		Lung cancer	374	31.2
K4	How long do you think is the biological half-life of bisphosphonates? (*n* = 1180)	Hours	8	0.7
		Days	18	1.5
		Weeks	81	6.9
		Months	263	22.3
		Years	810	68.6
M1	Does your medical history questionnaire inquire about the intake of antiresorptive drugs? (*n* = 1187)	Yes	828	69.8
M2	Do you offer a special recall program for edentulous patients who take drugs potentially causing osteonecrosis of the jaw? (*n* = 1185)	Yes	358	30.2
M3	Do you offer a special recall program for fully or partially dentate patients who take drugs potentially causing osteonecrosis of the jaw? (*n* = 1183)	Yes	503	42.5
M4	Which precautionary measures do you consider for tooth extractions in patients who take medication potentially causing osteonecrosis of the jaw? (*n* = 1197)	I do not take any precautionary measures	21	1.8
		I prescribe antibiotics 1–2 days before surgery	550	45.9
		I prescribe antibiotics postoperatively	122	10.2
		I smooth out bony spurs and close the wound area with a tension-free flap	558	46.6
		I do not treat such patients by myself	482	40.3

BRONJ: bisphosphonate-related osteonecrosis of the jaw; ARONJ: antiresorptive agent-related osteonecrosis of the jaw; ONJ: osteonecrosis of the jaw.

**Table 2:** Factors potentially influencing the outcome of the overall, knowledge, and management score. Significant *p*-values are highlighted in bold.

General Questions	Parameters	B	Standard Error	95% Wald Confidence Interval		Overall Score	Knowledge Score	Management Score
				Lower	Upper	Significance (*p*-Value)		
G1 (age group)	>65 years	−2.008	0.513	−3.013	−1.003	**<0.001**	**<0.001**	0.757
	56–65 years	−1.565	0.337	−2.226	−0.903	**<0.001**	**<0.001**	0.330
	46–55 years	−1.597	0.338	−2.260	−0.934	**<0.001**	**<0.001**	0.163
	36–45 years	−1.150	0.355	−1.847	−0.454	**0.001**	**0.001**	0.287
	<35 years (ref)	0						
G2 (place of work)	Hospital/dental clinic and dental office	0.680	0.505	−0.309	1.669	0.178	0.163	0.508
	Hospital or dental clinic	1.031	0.670	−0.282	2.344	0.124	0.313	0.387
	Dental office (ref)	0						
G3 (range of services)	Conservative dentistry and prosthetics	−0.080	0.405	−0.874	0.713	0.842	0.111	**0.036**
	Oral surgery	0.666	0.272	0.134	1.198	**0.014**	0.140	**0.003**
	Orthodontics	−0.090	0.275	−0.628	0.448	0.743	0.894	0.430
G4 (average of oral surgery)	>50%	3.131	0.717	1.726	4.535	**<0.001**	**0.003**	**<0.001**
	26–50%	1.254	0.681	−0.081	2.588	0.066	0.423	**0.013**
	6–25%	0.630	0.615	−0.575	1.835	0.305	0.738	0.140
	<5%	−0.216	0.599	−1.389	0.957	0.718	0.893	0.501
	0% (ref)	0						
G5 (continuous professional education)	Yes	1.997	0.233	1.540	2.454	**<0.001**	**<0.001**	**<0.001**
G6 (number of dental screening exams before antiresorptive therapy per year)	>15	3.804	0.461	2.900	4.708	**<0.001**	**<0.001**	**<0.001**
	11–15	2.213	0.535	1.165	3.260	**<0.001**	**0.004**	**<0.001**
	6–10	1.662	0.329	1.018	2.306	**<0.001**	**<0.001**	**<0.001**
	1–5	1.356	0.274	0.819	1.893	**<0.001**	**<0.001**	**0.003**
	0 (ref)	0						

Significant *p*-values are indicated in bold.

## Data Availability

Raw data will be made available upon reasonable request.
